# Marburgvirus in Egyptian Fruit Bats, Zambia

**DOI:** 10.3201/eid2508.190268

**Published:** 2019-08

**Authors:** Masahiro Kajihara, Bernard M. Hang’ombe, Katendi Changula, Hayato Harima, Mao Isono, Kosuke Okuya, Reiko Yoshida, Akina Mori-Kajihara, Yoshiki Eto, Yasuko Orba, Hirohito Ogawa, Yongjin Qiu, Hirofumi Sawa, Edgar Simulundu, Daniel Mwizabi, Musso Munyeme, David Squarre, Victor Mukonka, Aaron Mweene, Ayato Takada

**Affiliations:** Hokkaido University, Sapporo, Japan (M. Kajihara, H. Harima, M. Isono, K. Okuya, R. Yoshida, A. Mori-Kajihara, Y. Eto, Y. Orba, Y. Qiu, H. Sawa, A. Takada);; University of Zambia, Lusaka, Zambia (B.M. Hang’ombe, K. Changula, H. Sawa, E. Simulundu, M. Munyeme, A. Mweene, A. Takada);; Okayama University, Okayama, Japan (H. Ogawa);; Department of National Parks and Wildlife, Lusaka (D. Mwizabi, D. Squarre);; Zambia National Public Health Institute, Lusaka (V. Mukonka)

**Keywords:** Marburgvirus, Marburg virus disease, Filoviridae, Zambia, Egyptian fruit bats, Chiroptera, viruses

## Abstract

We detected Marburg virus genome in Egyptian fruit bats (*Rousettus aegyptiacus*) captured in Zambia in September 2018. The virus was closely related phylogenetically to the viruses that previously caused Marburg outbreaks in the Democratic Republic of the Congo. This finding demonstrates that Zambia is at risk for Marburg virus disease.

The genus *Marburgvirus*, like *Ebolavirus*, belongs to the family *Filoviridae* and consists of virus species that cause severe hemorrhagic fever in humans and nonhuman primates. *Marburgvirus* contains 1 species, *Marburg Marburgvirus*, and 2 viruses, Marburg virus (MARV) and Ravn virus (RAVV) (hereafter referred to as Marburgviruses) (*1*). Marburg virus disease (MVD) has occurred most frequently in central Africa countries such as Uganda and the Democratic Republic of the Congo (DRC) (*2*). Sporadic outbreaks including imported cases have also been reported in Angola, Kenya, and South Africa (*2*).

Epidemiologic evidence strongly suggests that Egyptian fruit bats (*Rousettus aegyptiacus*) are the primary natural reservoir of Marburgviruses. Entry into caves and mines inhabited by Egyptian fruit bats has frequently been linked to MVD outbreaks (*3*). Cave-dwelling Egyptian fruit bats in Uganda have been shown to maintain genetically diverse Marburgviruses for at least several years (*4**–**6*). However, key findings on Marburgvirus ecology have been obtained mainly through epidemiologic studies in endemic countries such as Uganda and the DRC. Although Egyptian fruit bats are widely distributed from Africa to the Middle East, northern India, and Pakistan (*7*), it remains elusive whether these bats outside endemic areas also harbor Marburgviruses.

Because a traveler who had visited Zimbabwe developed MVD in South Africa in 1975 (*3*), it has been suggested that countries in southern Africa are also at risk for MVD. Indeed, Angola has had the largest MVD outbreak, in 2004–2005 (*2*). To estimate the risk of MVD in Zambia, which has had no recognized human cases, we conducted an epidemiologic study of infection of Egyptian fruit bats with Marburgviruses in this country since 2014. Previously, we reported a high seroprevalence of Marburgvirus infection (43.8%) in the Egyptian fruit bat population in Zambia (*8*). Peaks of seroprevalence were repeatedly observed in November to December of each year, strongly suggesting the seasonality of infection in the Egyptian fruit bat colony in Zambia. However, neither infectious Marburgvirus nor its RNA genome had been detected in Egyptian fruit bats as of September 2018 (*8*).

In 2018, we captured 71 cave-dwelling Egyptian fruit bats in Lusaka Province, Zambia, as part of the research project Molecular and Serological Surveillance of Viral Zoonoses in Zambia (DNPW8/27/1), approved by the Department of National Parks and Wildlife, Ministry of Tourism and Arts of the Republic of Zambia (act no. 14 of 2015). We sampled lung, liver, kidney, spleen, and colon tissues from 22 bats in February and 25 bats in September (Table 1). In November, we collected oral and rectal swab samples from 24 bats. We extracted total RNA from pooled tissue homogenates (lung, liver, kidney, and spleen), colon homogenates, and pooled swab samples, as described previously (*8*). Subsequently, we tested RNA samples by reverse transcription PCR with panfilovirus nucleoprotein (NP) (*9*), Marburgvirus NP, viral protein (VP) 35 (*6*), and RNA-dependent RNA polymerase (L) gene primer sets (*10*).

**Table 1:** Summary of Egyptian fruit bats (*Rousettus aegyptiacus*) captured in 2018 in Zambia and genetic screening results

Sampling month	No. tested bats (sex)	RNA source	No. (%) positive
Feb	22 (10 M, 12 F)	Tissue pool,* colon	0 (0)
Sep	25 (13 M, 12 F)	Tissue pool,* colon	2 (8.0)
Nov	24 (1 M, 23 F)	Swab pool†	0 (0)

*Liver, lung, kidney, and spleen were pooled for each bat. †Oral and rectal swabs were pooled for each bat.

We obtained all the expected PCR products from the RNA samples of an Egyptian fruit bat (ZB18-36) captured in September (Table 2). By Sanger sequencing of the PCR products and subsequent BLAST searches (https://blast.ncbi.nlm.nih.gov/Blast.cgi), we confirmed detection of Marburgvirus NP, VP35, and L genes (GenBank accession nos. LC465155–7). We also detected the NP gene in the pooled tissue RNA of another bat (ZB18-55) (GenBank accession no. LC465158) (Table 2). Nucleotide sequences of these genes were highly similar to those of the viruses that caused outbreaks in DRC during 1998–2000. Pairwise comparison of partial NP (560-nt), VP35 (344-nt), and L (292-nt) gene sequences showed 99.1%, 96.8%, and 99.7% identities, respectively, to the respective genes of strain MARV/H.sapiens-tc/COD/2000/25 DRC (GenBank accession no. JX458849). We excluded the possibility of laboratory contamination because the detected sequences were distinct from those of viral RNA or plasmids containing Marburg virus genes used in our laboratory.

**Table 2:** Summary of Egyptian fruit bats (*Rousettus aegyptiacus*) positive for Marburgviruses by reverse transcription PCR, Zambia, 2018*

Bat ID	Sex	Body weight, g	Sample	Reverse transcription PCR primer set			
Filo NP	Marburg NP	Marburg VP35	Marburg L
ZB18-36	F	80	Tissue pool†	–	+	–	+
			Colon	+	+	+	+
ZB18-55	M	100	Tissue pool†	–	+	–	–
			Colon	–	–	–	–

*L, RNA-dependent RNA polymerase; NP, nucleoprotein; VP35, viral protein 35; –, negative; +, positive. †Liver, lung, kidney, and spleen were pooled.

Subsequently, we phylogenetically compared the NP, VP35, and L genes with representative Marburgvirus sequences available in GenBank. We aligned nucleotide sequences by MUSCLE (https://www.ebi.ac.uk/Tools/msa/muscle) and constructed phylogenetic trees by the maximum-likelihood method with 1,000 bootstrap replicates in MEGA7 (*11*). The NP, VP35, and L-based trees showed similar topology and clearly demonstrated that the virus in Zambia belonged to the MARV, not the RAVV, lineage (Figure). The virus was closely related to Marburgviruses detected in Egyptian fruit bats in Uganda (*5**,**6*), as well as to those that caused human cases in the DRC, but were phylogenetically distinct from the viruses that caused MVD in Angola.

**Figure:**
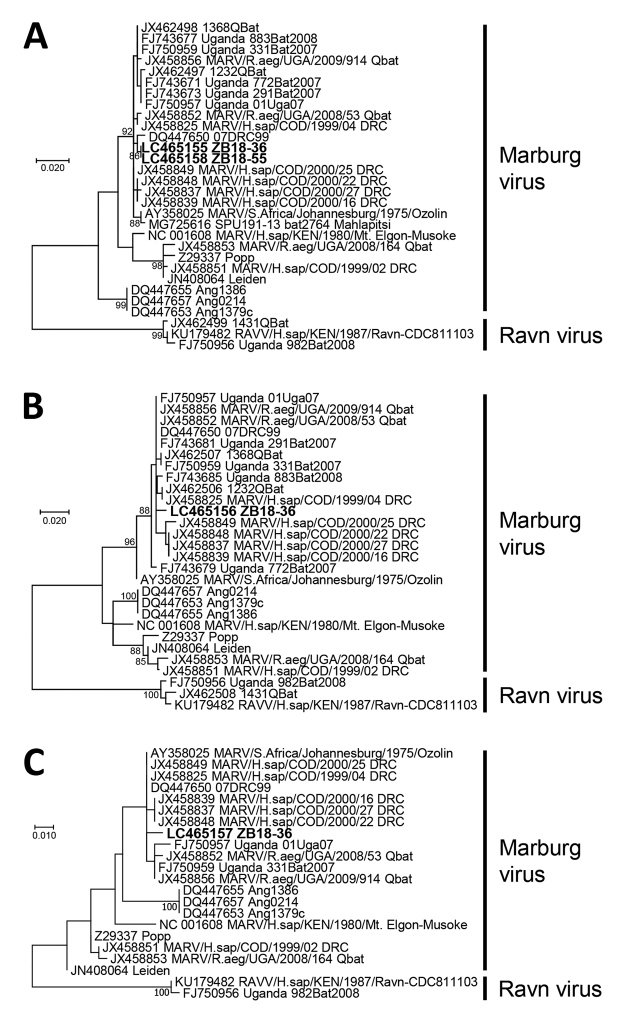
Phylogenetic trees showing evolutionary relationships of Marburgviruses from Egyptian fruit bats (*Rousettus aegyptiacus*), Zambia, 2018 (boldface), and reference viruses. The trees were constructed based on nucleotide sequences of 440 nt for the nucleoprotein gene (A), 296 nt for the viral protein 35 gene (B), and 238 nt for the RNA-dependent RNA polymerase gene (C) by using the maximum-likelihood method in MEGA7 (*11*). Nucleotide sequences of representative Marburgvirus strains were obtained from GenBank; accession numbers are shown with strain names. Bootstrap values >80 are shown near the branch nodes. Scale bars indicate nucleotide substitutions per site.

Recently, the Marburgvirus genome was detected in cave-dwelling Egyptian fruit bats in South Africa (*10*), suggesting that this virus might be maintained by bats in southern Africa, including previously nonendemic countries. Our previous serologic data also indicate that Marburgviruses may actively circulate in the Egyptian fruit bat population in this region, including Zambia, rather than being introduced occasionally from endemic areas in 2018, as the seroprevalence of Marburgvirus infection among Egyptian fruit bats was repeatedly increased in November–December (*8*). Furthermore, the viruses detected in Zambia, Uganda, the DRC, and South Africa belong to the same cluster phylogenetically. Taken together, these findings suggest that Marburgviruses may be maintained by the larger metapopulation of Egyptian fruit bats distributed in sub-Saharan Africa. Egyptian fruit bats are known to migrate several hundred kilometers (*7*), and the Marburgvirus genome has also been detected in *Miniopterus inflatus* and *Rhinolophus eloquens* bats, as well as Egyptian fruit bats in Gabon (*4*). Frequent contacts among multiple species of bats via long-distance movement may facilitate the maintenance of genetically diverse Marburgviruses in African bats.

Experimental infection of Egyptian fruit bats with MARV demonstrated that induced virus-specific IgG rapidly declined by 3 months postinfection (*12*). Considering the serologic peaks in November to December in the Egyptian fruit bat population in Zambia (*8*), it is reasonable that the virus was detected in September in this study. The prevalence of Marburgvirus infection in the bat colony has probably decreased to an undetectable level as the seroprevalence in the bats increases. Previous studies have suggested that biannual reproduction of Egyptian fruit bats in Uganda provides appropriate conditions for Marburgvirus perpetuation relying on the increased population of susceptible juvenile bats associated with the decline of maternal antibodies (*6**,**13*). However, this transmission manner may not be the case for Egyptian fruit bats in southern Africa, as they give birth once a year (*7**,**8**,**10*). Recently, Schuh et al. demonstrated that Marburgvirus was horizontally transmitted from inoculated to contact Egyptian fruit bats even at 7 months postinfection (*12*), suggesting that the viruses could establish persistent infection in this bat species. Even in humans, long-term viral persistence in immune-privileged sites such as the testes and eyes has occasionally been reported (*14*). Because 42% of the female bats captured in September (5 of 12 bats) were pregnant, it could still be assumed that seasonal biologic events such as breeding might be a possible trigger for recurrence of Marburgvirus infection in the bat colonies.

We report a potential risk for MVD in Zambia. It is important to clarify whether unrecognized human cases of MVD, including asymptomatic Marburgvirus infection, are present in Zambia. Moreover, further research is needed to elucidate the ecology of Marburgviruses in the entire African region and to estimate potential risks of MVD outbreaks in previously nonendemic countries. In particular, more extensive information is needed on Marburgviruses in the Egyptian fruit bat population, including the genetic diversity of the viruses, the distribution and migratory behavior of the bats, and the seasonal pattern of virus infection prevalence.

## References

[1] Amarasinghe GK, Aréchiga Ceballos NG, Banyard AC, Basler CF, Bavari S, Bennett AJ, et al. Taxonomy of the order Mononegavirales: update 2018. Arch Virol. 2018;163:2283–94. 10.1007/s00705-018-3814-x 29637429 PMC6076851

[2] Centers for Disease Control and Prevention. Outbreaks chronology: Marburg hemorrhagic fever. 2019 [cited 2019 Jan 29]. https://www.cdc.gov/vhf/marburg/outbreaks/chronology.html

[3] Changula K, Kajihara M, Mweene AS, Takada A. Ebola and Marburg virus diseases in Africa: increased risk of outbreaks in previously unaffected areas? Microbiol Immunol. 2014;58:483–91. 10.1111/1348-0421.12181 25040642

[4] Swanepoel R, Smit SB, Rollin PE, Formenty P, Leman PA, Kemp A, et al.; International Scientific and Technical Committee for Marburg Hemorrhagic Fever Control in the Democratic Republic of Congo. Studies of reservoir hosts for Marburg virus. Emerg Infect Dis. 2007;13:1847–51. 10.3201/eid1312.071115 18258034 PMC2876776

[5] Towner JS, Amman BR, Sealy TK, Carroll SA, Comer JA, Kemp A, et al. Isolation of genetically diverse Marburg viruses from Egyptian fruit bats. PLoS Pathog. 2009;5:e1000536. 10.1371/journal.ppat.1000536 19649327 PMC2713404

[6] Amman BR, Carroll SA, Reed ZD, Sealy TK, Balinandi S, Swanepoel R, et al. Seasonal pulses of Marburg virus circulation in juvenile *Rousettus aegyptiacus* bats coincide with periods of increased risk of human infection. PLoS Pathog. 2012;8:e1002877. 10.1371/journal.ppat.1002877 23055920 PMC3464226

[7] AfricanBats NPC, African Chiroptera Project. African Chiroptera Report 2018. Pretoria (South Africa): AfricanBats NPC; 2018.

[8] Changula K, Kajihara M, Mori-Kajihara A, Eto Y, Miyamoto H, Yoshida R, et al. Seroprevalence of filovirus infection of *Rousettus aegyptiacus* bats in Zambia. J Infect Dis. 2018;218(suppl5):S312–7. 29889270 10.1093/infdis/jiy266

[9] Ogawa H, Miyamoto H, Ebihara H, Ito K, Morikawa S, Feldmann H, et al. Detection of all known filovirus species by reverse transcription-polymerase chain reaction using a primer set specific for the viral nucleoprotein gene. J Virol Methods. 2011;171:310–3. 10.1016/j.jviromet.2010.11.010 21093485 PMC3393132

[10] Pawęska JT, Jansen van Vuren P, Kemp A, Storm N, Grobbelaar AA, Wiley MR, et al. Marburg virus infection in Egyptian rousette bats, South Africa, 2013–2014. Emerg Infect Dis. 2018;24:1134–7. 10.3201/eid2406.172165 29774854 PMC6004853

[11] Kumar S, Stecher G, Tamura K. MEGA7: Molecular Evolutionary Genetics Analysis version 7.0 for bigger datasets. Mol Biol Evol. 2016;33:1870–4. 10.1093/molbev/msw054 27004904 PMC8210823

[12] Schuh AJ, Amman BR, Jones ME, Sealy TK, Uebelhoer LS, Spengler JR, et al. Modelling filovirus maintenance in nature by experimental transmission of Marburg virus between Egyptian rousette bats. Nat Commun. 2017;8:14446. 10.1038/ncomms14446 28194016 PMC5316840

[13] Hayman DTS. Biannual birth pulses allow filoviruses to persist in bat populations. Proc Biol Sci. 2015;282:20142591. 25673678 10.1098/rspb.2014.2591 PMC4345444

[14] Schindell BG, Webb AL, Kindrachuk J. Persistence and sexual transmission of filoviruses. Viruses. 2018;10:E683. 10.3390/v10120683 30513823 PMC6316729

